# Na_4_Mn_9_O_18_/Carbon Nanotube Composite as a High Electrochemical Performance Material for Aqueous Sodium-Ion Batteries

**DOI:** 10.1186/s11671-017-2340-1

**Published:** 2017-10-17

**Authors:** Fuxing Yin, Zhengjun Liu, Shuang Yang, Zhenzhen Shan, Yan Zhao, Yuting Feng, Chengwei Zhang, Zhumabay Bakenov

**Affiliations:** 1 School of Materials Science & Engineering, Research Institute for Energy Equipment Materials, Tianjin Key Laboratory of Materials Laminating Fabrication and Interface Control Technology, Hebei University of Technology, Tianjin, 300130 China; 2 Synergy Innovation Institute of GDUT, Heyuan, Guangdong Province China; 3 School of Engineering, Nazarbayev University, Kabanbay Batyr Ave. 53, Astana, Kazakhstan 010000

**Keywords:** Aqueous sodium-ion battery, Cathode, Na_4_Mn_9_O_18_/carbon nanotube, Energy storage and conversion

## Abstract

The aqueous sodium-ion battery (ASIB) is one of the promising new energy storage systems owing to the abundant resources of sodium as well as efficiency and safety of electrolyte. Herein, we report an ASIB system with Na_4_Mn_9_O_18_/carbon nanotube (NMO/CNT) as cathode, metal Zn as anode and a novel Na^+^/Zn^2+^ mixed ion as electrolyte. The NMO/CNT with microspherical structure is prepared by a simple spray-drying method. The prepared battery delivers a high reversible specific capacity and stable cyclability. Furthermore, the battery displays a stable reversible discharge capacity of 53.2 mAh g^−1^ even at a high current rate of 4 C after 150 cycles. Our results confirm that the NMO/CNT composite is a promising electrode cathode material for ASIBs.

## Background

Lithium-ion batteries (LIBs) are regarded as promising power sources for their applications in portable electronic devices [1, 2]. Specifically, various aqueous LIB systems have been attracting growing attention due to their low cost, safety, and high-rate capability [3]. In 1994, Dahn group for the first time proposed an aqueous LIB system [4]. Since then, lots of electrode materials such as LiFePO_4_ [5], LiMn_2_O_4_ [6], and LiCoO_2_ [7] have been developed. Nevertheless, the previous reports focus on the lithium materials as anode. However, lithium has low abundance in the earth’s crust, which could potentially increase costs and curtail large-scale implementation [8].

The aqueous sodium-ion batteries (ASIBs) have gained much attention as substitutes for aqueous LIBs due to the good chemical properties and low prices of the sodium. Presently, various sodium-based materials such as Na_3_V_2_(PO_4_)_3_ [9], Na_2_FeP_2_O_7_ [10], Na_2_CuFe(CN)_6_ [11], and Na_4_Mn_9_O_18_ have been developed as active materials for ASIBs [12]. Among them, Na_4_Mn_9_O_18_ (NMO) with orthogonal structure has two types of tunnels (S-type and O-type) formed by the MnO_6_ octahedra and MnO_5_ square pyramids. Two sodium sites occupy the large S-type tunnels as well as one sodium site occupies smaller O-type tunnels [13]. These tunnels in NMO are conducive to the transmission of sodium ions. However, low conductivity and enormous volume expansion of the NMO during the insertion/extraction of large Na^+^ resulting in the pulverization of the active material hinder its application as cathode in ASIBs. [14–16]. Therefore, various approaches have been developed to solve the abovementioned issues. One of the effective methods is to composite with carbon materials with great conductive and chemical stability that can buffer the volume change, simultaneously enhancing the electrode’s electrical conductivity [17, 18]. Nowadays, nanostructured carbon materials such as porous carbon [19], spherical carbon [20], graphene [21], and carbon nanotube [3, 22] are widely used as active material supports. Among the various substrates, carbon nanotube could form a conductive skeleton, which can increase the transmission path of electrons and enhance the mechanical properties of the material [23]. Furthermore, the structure of the cathode material such as particle size, microscopic morphology, and specific surface area seriously affect the electrochemical performance of the battery [24]. Compared to irregular powders, microspherical powders with narrow size distributions exhibit great electrochemical properties due to their high tap-density. Spray-drying is an effective route to synthesize fine multi-component and homogeneous spherical powders [25]. The composite samples with microspherical structure such as LiFePO_4_/C and Li_4_Ti_5_O_12_/C [24, 26] have been synthesized by spray-drying and are used in lithium-ion batteries, but spray-drying techniques are rarely used in sodium ion batteries.

**Fig. 1:**
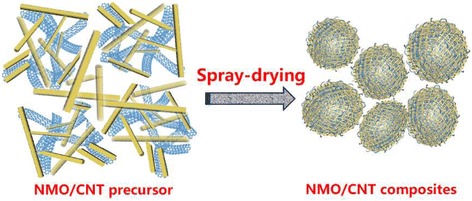
The schematic diagram of NMO/CNT

In this work, we for the first time report the preparation of Na_4_Mn_9_O_18_/carbon nanotube (NMO/CNT) composite with microspherical structure via the spray-drying method (Fig. 1). The ASIBs were then constructed with NMO/CNT as a cathode, novel Na^+^/Zn^2+^ mixed-ion as electrolytes, and zinc metal as anode. In this novel battery system, the electrochemical properties of the resulting NMO/CNT composite as the cathode have been investigated.

## Methods

### Material Preparation

The NMO/CNT composite precursor was firstly prepared as following: 4.0 mg of CNT aqueous dispersion (9 wt%, Timesnano, Chengdu) was added into 30 mL of 0.1 M KMnO_4_ and 3.0 M NaOH aqueous solution under stirring. Then, 30 mL of 0.28 M MnSO_4_ solution was dropped into the above mixed solution, and the brown precipitation was produced suddenly. The resulting precipitation was obtained by centrifugal method and allowed to stand for 24 h to form wet-aged sample. Next, 4 g of wet-aged sample was added to 100 mL of 15 M NaOH solution and stirred for 25 min to form the dark brown suspension. Finally, the suspension was heated at 180 °C for 24 h using a 150-mL stainless steel autoclave with a Teflon liner. The NMO/CNT composite precursor was washed repeatedly with deionized water and dried at 80 °C in the air.

To prepare the NMO/CNT composite, 0.6 g of NMO/CNT precursor was added into 150 mL deionized water with ultra-sonication for 15 min to form a brown suspension. The suspension was added into the spray-drying machine (HOLVES, Beijing) by peristaltic pump at 6 mL min^−1^. Then, it was atomized at 205 °C applying a two-fluid nozzle with atomizing pressure of 0.8 MPa and the outlet temperature of 110 °C. The obtained powder is the desired NMO/CNT composite with microspherical structure. The reference NMO particle without CNT was prepared following the same conditions.

### Material Characterization

X-ray powder diffraction (XRD) date of the prepared samples was measured by an X-ray diffraction (XRD, D8 Discover, Bruker) employing Cu Kα radiation. Thermo-gravimetric (TG, SDT Q-600, TA Instruments-Waters LLC) analysis was carried out from 25 to 1000 °C with a heating rate of 10 °C min^−1^ under air. Raman spectroscopy was carried out using a Jobin-Yvon T6400 Micro-Raman system with a 532-nm argon-ion laser. Scanning electron microscopy (SEM) analysis was collected on a Hitachi Limited S-4800 scanning electron microscope. The interior structure and selected area electron diffraction (SAED) of samples were studied using a JEOL JEM-2800 high-resolution transmission electron microscope (HR-TEM) at 160 kV. The Mn content in the electrolyte was measured by inductive coupled plasma optical emission spectroscopy (ICP-OES, PRODIGY XP, LEEMON).

### Electrochemical Measurements

To prepare the NMO/CNT composite electrode, the slurry was first prepared by mixing with 80 wt% as-prepared sample, 10 wt% acetylene black, and 10 wt% polyvinylidene fluoride (PVDF) in *N*-methyl-2-pyrrolidone (NMP). The above slurry was spread uniformly onto a carbon foil current collector and dried at 75 °C for 12 h. The above carbon foil and Zn metal foil were cut into circular disks with 15 mm in diameter as the cathode and anode, respectively. The solution containing 1 M Na_2_SO_4_ and 0.5 M ZnSO_4_ with the pH = 4 was used as the electrolyte, and absorbed glass mat (NSG Corporation) was applied as separator [27, 28]. 2025 coin-type batteries were assembled in air atmosphere before electrochemical tests. The charge/discharge cycling performance was investigated on a battery testing system (Neware, Shenzhen) in the potential range of 1–1.85 V (vs. Zn^2+^/Zn). Cyclic voltammetries (CVs) were carried out by the electrochemical workstation (Princeton, VersaSTAT 4) in the potential range of 1–2 V (vs. Zn^2+^/Zn). The electrochemical impedance spectroscopy (EIS) was performed by using the electrochemical workstation (Princeton, VersaSTAT 4) in the frequency range of 0.01–100 kHz.

## Results and Discussion

**Fig. 2:**
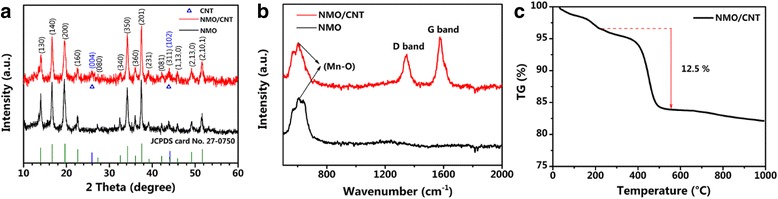
**a** XRD patterns of NMO/CNT and NMO. **b** Raman spectrum of NMO/CNT and NMO. **c** TG curves of the NMO/CNT composite at a heating rate of 10 °C/min under air

The XRD patterns of the NMO/CNT and NMO are shown in Fig. 2a. Both XRD patterns agree well with the phase of NMO (JCPDS #27-0750) [29], revealing that hydrothermal and spray-drying method is a valid route to synthesis the Na_4_Mn_9_O_18_-based materials. The XRD pattern of NMO/CNT exhibits two broadened peaks at ca. 26° and 44° in NMO/CNT composite, which are associated with the graphitic planes (004) and (102) of the CNT, respectively [30, 31], suggesting that the NMO/CNT composite is successfully prepared.

Figure 2b shows the Raman spectra of the NMO/CNT composite and the pure NMO sample. The broad peaks at around 600–650 cm^−1^ may be related to stretching vibrations of Mn–O band [23]. The spectrum of the NMO/CNT shows two broad and strong peaks located at about 1347 cm^−1^ (D-band) and 1575 cm^−1^ (G-band), corresponding to the graphitic carbon in CNT [32, 33]. The Raman results confirm that the as-prepared NMO/CNT consists of pure CNT and crystalline NMO nanoparticles. The actual content of CNT in NMO/CNT composite was measured by TG analysis in air flux. As shown in Fig. 2c, the first weight loss of the NMO/CNT under 220 °C is caused by the evaporation of adsorbed water molecules. The following major weight loss occurring from 350 to 500 °C is related to the CNT combustion. Based on the TG curve of the NMO/CNT composite, the actual NMO content in the composite is calculated to be 87 wt%.

**Fig. 3:**
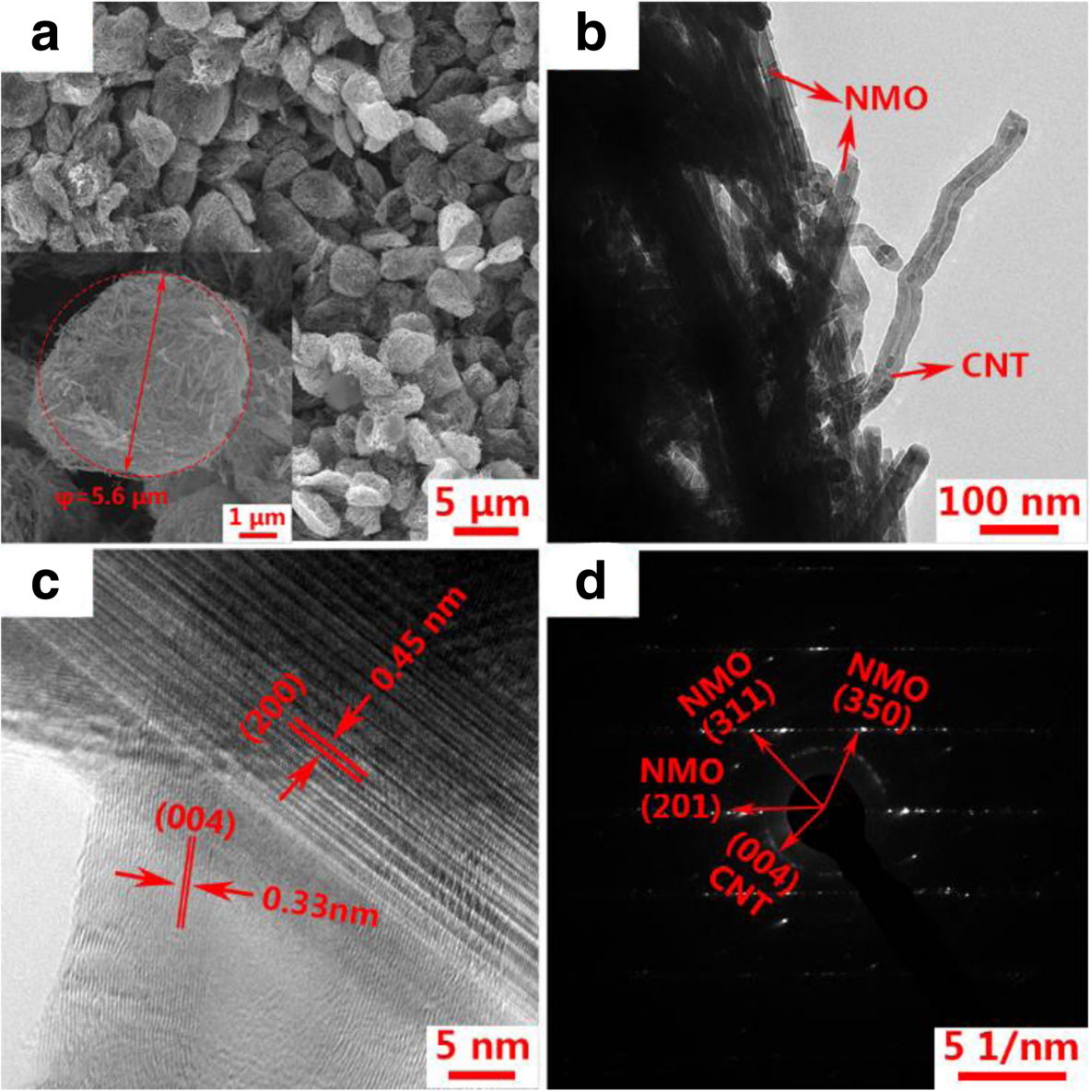
The morphology and structure of the NMO/CNT. **a** SEM image; **b** TEM image; **c** HRTEM image; **d** SAED pattern

SEM and TEM were used to determine the morphologies of the samples. Figure 3a shows the SEM image of the NMO/CNT composite where uniform microspheres with diameter of about 5–7 μm can be observed. One of the spheres is enlarged in the inset of Fig. 3a, and intertwined CNT can be observed. The CNT networks are very important because they can capture NMO of the rod structure. Figure 3b displays a TEM image of the NMO/CNT composite; more clearly, the crosslinking state of NMO/CNT can be observed. The rod-shaped NMOs with diameter of around 30–50 nm are wound together by CNT that can enhance the electrical conductivity of the composite cathode material. Figure 3c shows the lattice fringes with an inter-fringe distance of 0.45 and 0.33 nm, corresponding to (200) of NMO and (004) of CNT, respectively. The SAED pattern (Fig. 3d) exhibits the single crystal nature of NMO, indicating a high crystallinity of NMO. And the homogeneous diffraction rings of CNT also can be observed from SAED pattern, which confirms that the NMO is successfully composited with CNT by a simple spray-drying method.

**Fig. 4:**
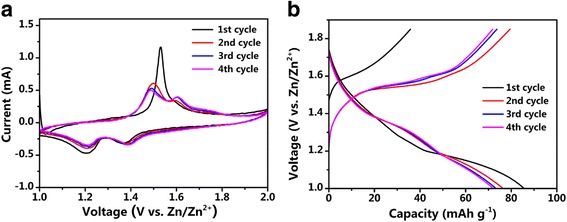
Electrochemical performance of NMO/CNT electrode. **(a)** CV behavior of NMO/CNT electrode at a scan rate of 0.1 mV s^−1^. **(b)** Discharge/charge voltage profiles of NMO/CNT electrode for the 1st, 2nd, 3rd, and 4th cycles at 4 C

Figure 4a shows the CV curves of the cell with NMO/CNT composite as cathode at a san rate of 0.1 mV s^−1^. The CV curves show two reduction peaks at around 1.20 and 1.37 V (vs. Zn^2+^/Zn) as well as one oxidation peak at about 1.53 V (vs. Zn^2+^/Zn) during the initial cycle. The oxidation peak in the initial cycle shows one higher peak current compared to the rest of the scans/cycles, which could be due to initial multi-atomic phase transitions to adapt the strain when the Na ion was extracted from the NMO/CNT materials, indicating some degree of irreversibility in the first cycle [34]. For the following cycles, the data displays two main redox couples at about 1.50/1.20 V and 1.62/1.37 V (vs. Zn^2+^/Zn), respectively. They are associated with the de-insertion and insertion of Na ions from/into the orthorhombic crystal structure of NMO in the aqueous electrolyte. During the charge process, Na ions are extracted from NMO/CNT cathode to the electrolyte, which is accompanied by liberation of the electrons while Zn ions are deposited on the surface of zinc anode. When battery is discharged, zinc of anode loses the electrons and dissolves to the electrolyte, while at the cathode, the reaction is reversed and Na ions are inserted into cathode to form NMO/CNT [35]. These reactions could be represented as follows:

$$ {\displaystyle \begin{array}{l}\mathrm{anode}\  \mathrm{reaction}:{\mathrm{Zn}}^{2+}+2{\mathrm{e}}^{-}\iff \mathrm{Zn}\\ {}\mathrm{cathode}\  \mathrm{reaction}:{\mathrm{Na}}_4{\mathrm{Mn}}_9{\mathrm{O}}_{18}\iff {\mathrm{Na}}_{4-x}{\mathrm{Mn}}_9{\mathrm{O}}_{18}+x{\mathrm{Na}}^{+}+x{\mathrm{e}}^{-}\\ {}\mathrm{total}\  \mathrm{reaction}:2{\mathrm{Na}}_4{\mathrm{Mn}}_9{\mathrm{O}}_{18}+x{\mathrm{Zn}}^{2+}\iff 2{\mathrm{Na}}_{4-x}{\mathrm{Mn}}_9{\mathrm{O}}_{18}+2x{\mathrm{Na}}^{+}+x\mathrm{Zn}\end{array}} $$

The weak oxidation peak at about 2 V (vs. Zn^2+^/Zn) is related to decomposition of water in the electrolyte [27]. With increasing cycles, the potentials of the redox peak tend to be stable. The symmetrical peaks demonstrate that the Na ion de-insertion/insertion process can be regarded as highly reversible. It has been reported that for the NMO materials, the capacity decay will arise from the continuous dissolution of Mn^2+^ upon a disproportionation reaction of Mn^3+^ into Mn^2+^ and Mn^4+^ [29]. In order to evaluate the effect of this phenomenon in our battery, the Mn ion content in the electrolyte was measured by ICP-OES. The concentration of Mn ions in 25 mL of electrolyte in contact with 0.12 g of active material was measured under different acidic environments as shown in Table 1.

**Table 1:** Concentration of Mn ions in Na^+^/Zn^2+^ mixed-ion electrolyte after exposure to Na_4_Mn_9_O_18_ for 48 h

Electrolyte pH	1	2.5	4
Mn concentration (μg/mL)	76.376	40.263	12.455

The results of this experiment showed that the content of Mn ions decreased with the increase of pH. Therefore, the pH of the electrolyte was set to 4 [36], which inhibits the disproportionation reaction mentioned above and reduces the Mn dissolution.

The galvanostatic discharge/charge was conducted to measure the Na storage capability. From the CV curve, we learned that the voltage should be less than 2 V to prevent the water decomposition reaction. Figure 4b illustrates the discharge/charge curves of the NMO/CNT electrode during the initial four cycles at 4 C in the potential range of 1–1.85 V (vs. Zn^2+^/Zn). In the first cycle, the charge capacity is only 35.8 mAh g^−1^ and considerable corresponding discharge capacity is 85.6 mAh g^−1^. Thus, the coulombic efficiency at the initial cycle is 239.4%, much larger than 100%, which is due to the decomposition of interstitial water and low content of Na ion [37]. After the first cycle, the potential plateaus were well-maintained upon further cycling. There are two conspicuous potential plateaus at around 1.37 and 1.2 V (vs. Zn^2+^/Zn) in the discharge curves as well as two plateaus at about 1.50 and 1.62 V (vs. Zn^2+^/Zn), which corresponds to the 2nd, 3rd, and 4th CV cycles.

**Fig. 5:**
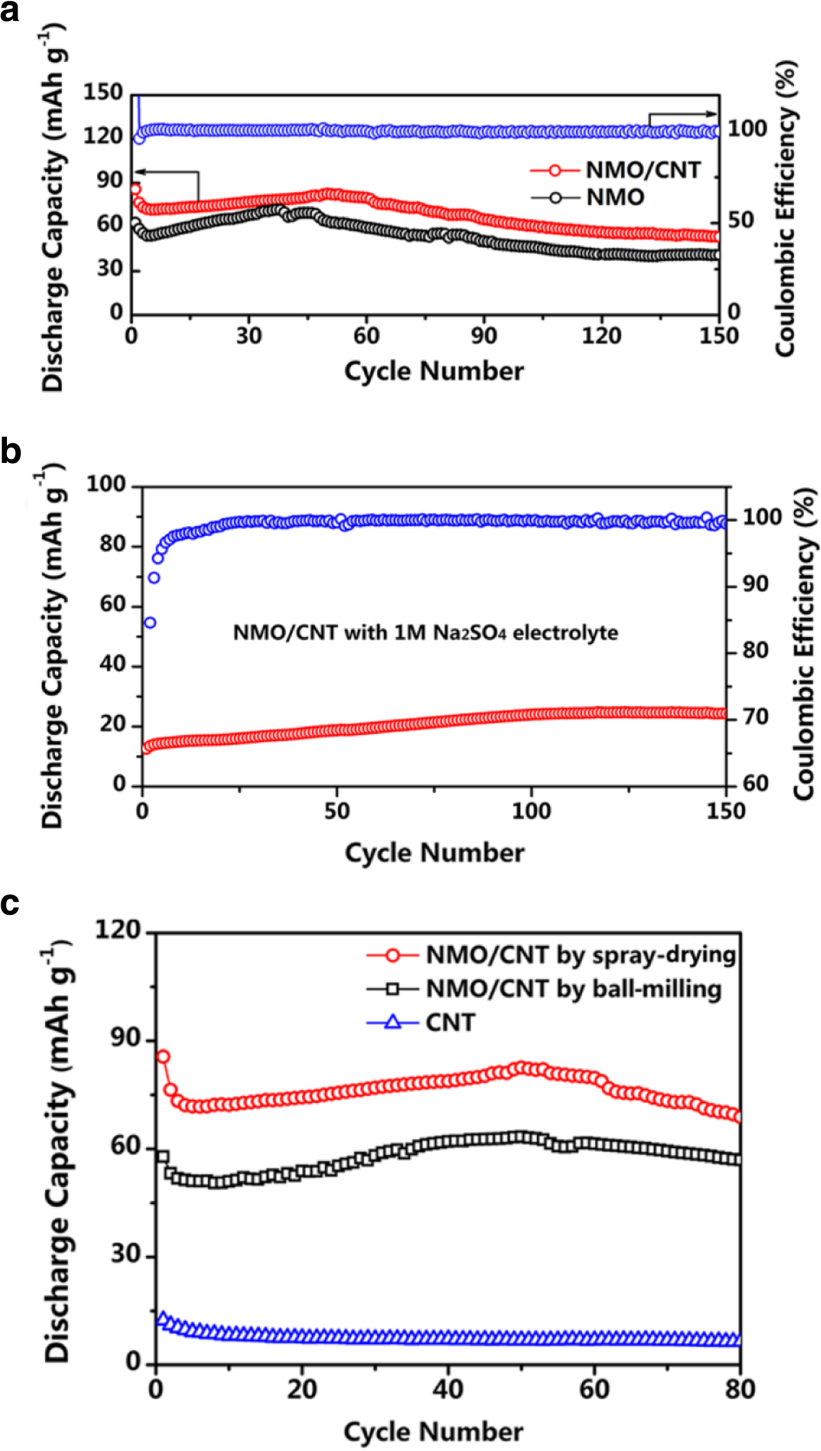
Cycling performances at 4 C. **a** Cycling performance comparison of NMO and NMO/CNT electrodes and coulombic efficiency of NMO/CNT electrode. **b** Cycling performance and coulombic efficiency of NMO/CNT electrode with 1 M Na_2_SO_4_ electrolyte. **c** Cycling performance comparison of NMO/CNT electrodes via the spray-drying method and ball-milling method, respectively

Figure 5a presents the cycling performance of NMO and NMO/CNT electrodes at 4 C rate. It can be seen that the initial discharge capacities of NMO and NMO/CNT are as high as 62.7 and 85.6 mAh g^−1^, respectively. The capacities of the two different electrodes dropped rapidly in first few cycles, which are related to the irreversible reaction in the initial cycle. Then, the capacities of the electrodes are stabilized for the continuous cycles. The coulombic efficiency of the NMO/CNT electrode gradually approaches 100% and keeps steady during the following cycles. For the NMO/CNT electrode, it delivers a reversible capacity of 53.2 mAh g^−1^ after 150 cycles, better than that of the NMO electrode (40 mAh g^−1^). The high discharge specific capacity may arise from the introduction of CNT electronic transmission network, which promotes charge transfer and phase evolution. Furthermore, the CNT could buffer stress and strain upon Na^+^ ion de-insertion/insertion due to that they are flexible [23]. In addition, the spherical structure of the NMO/CNT can provide the rapid diffusion of the Na ion and electrolyte. The electrochemical performance enhancement of NMO/CNT may also be attributed to the use of a suitable electrolyte. For comparison purposes, a cell was assembled with 1 M Na_2_SO_4_ aqueous solution as the electrolyte. This cell delivered a reversible discharge capacity of only around 24 mAh g^−1^ after 150 cycles (Fig. 5b), i.e., the reversible capacity of the system has been significantly reduced when there is no ZnSO_4_ in the electrolyte.

In order to illustrate a positive effect of morphology of the NMO/CNT composite on its electrochemical performances, non-spherical NMO/CNT composite was synthesized via a simple ball-milling method. NMO and CNT with a weight ratio of 87:13 were mixed by ball-milling at 400 rpm for 6 h to obtain this reference composite material. The electrochemical performance of this composite is shown in Fig. 5c along with that of spherical NMO/CNT composite by spray-drying. It can be seen that non-spherical NMO/CNT exhibits remarkably lower discharge capacities compared with the spherical counterpart, NMO/CNT composite by spray-drying. In Fig. 5c, the electrochemical data for pure CNT were included to evaluate its contribution to the cell capacity. CNT delivers a small reversible capacity of 6.5 mAh g^−1^ at 4 C and does not remarkably contribute to the total capacity, and its main role is to increase the composite conductivity and support its mechanical properties.

**Fig. 6:**
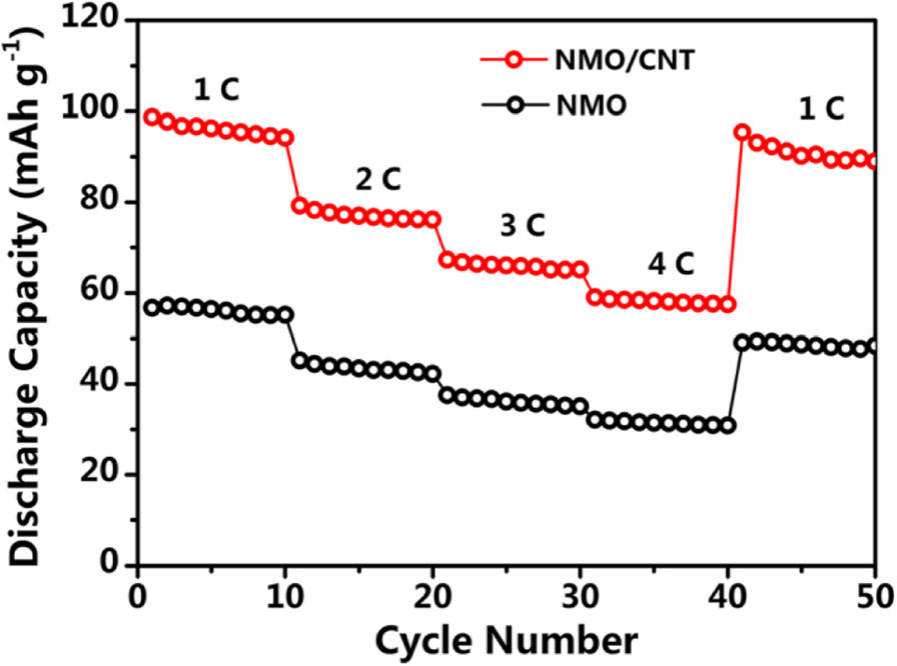
Rate performances of NMO and NMO/CNT electrodes from 1 to 4 C

To demonstrate the rate performance of the NMO/CNT composite, the rate capabilities were tested at various current densities from 1 to 4 C. Figure 6 shows that the NMO/CNT composite delivers a reversible discharge capacity of 96, 77, 66, and 58 mAh g^−1^ at the current of 1, 2, 3, and 4 C, respectively. Importantly, after the high-current-density measurements, once the current density returned to 1 C, the specific capacity was almost reverted to the same level. As can be seen, the reversible discharge capacities of the NMO at each current rate were lower than that of the NMO/CNT, but the electrode also regained closely full of its reversible capacity 50 mAh g^−1^ when the rate was modulated back to 1 C. These results not only illustrate that the CNT of the NMO/CNT composite has enhanced reversible capacity, but also indicate that the NMO material has the high abuse tolerance that can exhibit excellent rate performance. The outstanding rate performance of NMO and NMO/CNT composite may be due to the microspherical structure that can enhance the mass transport of the Na ion and electrolyte.

**Fig. 7:**
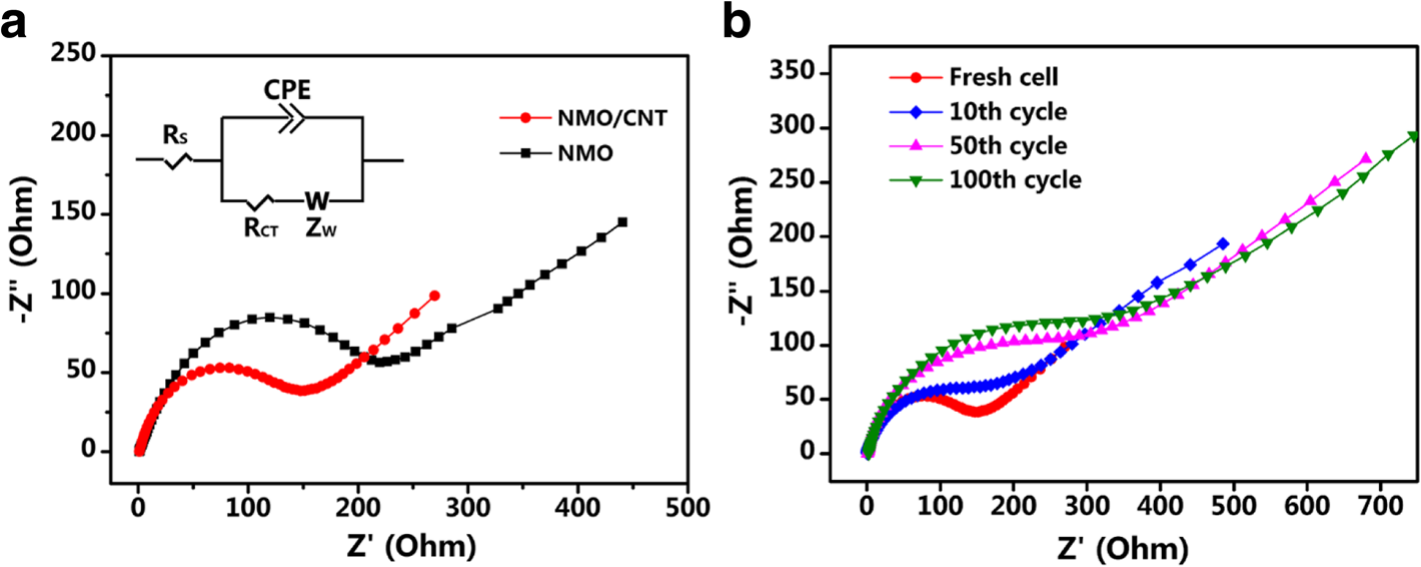
**(a)** EIS spectra of NMO and NMO/CNT electrodes at the fresh cycle and the equivalent circuit model of plot fitting (*inset*). **(b)** EIS spectra of NMO/CNT electrode at the fresh cell, 10th, 50th, and 100th cycles

Electrochemical impedance spectroscopy (EIS) of the NMO and NMO/CNT electrodes measured with the frequency range of 10^5^–0.01 Hz are displayed in Fig. 7. The inset of Fig. 7a is a simple equivalent circuit model applied to fit the EIS. The *R*_S_ is the electrolyte resistance of cell components, *R*_CT_ is related to the charge transfer procedure at the electrode-electrolyte interface, *Z*_W_ is the Warburg impedance that associated with sodium-ion diffusion in the electrode, and CPE is associated with the double-layer capacitance [38]. As shown in Fig. 7a, the charge transfer resistance (*R*_CT_) of the NMO/CNT electrode is 133 Ω, and the value is significantly smaller than that of NMO (207 Ω), demonstrating that the CNT are beneficial for the enhancement of the electrochemical performance of NMO/CNT electrode. In addition, the resistance of NMO/CNT gradually increases with the progress of the cycle as shown in Fig. 7b, which is related to the internal activation of the electrode, corresponding to the cycling performance image (Fig. 5a). EIS spectra of the 50th and 100th cycles are almost identical, which suggests that the resistances are relatively stable as the cycle progresses.

Table 2 compares the performance data reported for the Na_*x*_MnO_2_-based cathodes for aqueous sodium-ion batteries. It can be observed that the NMO/CNT electrode prepared in this work displays superior electrochemical performance compared with those reported. At a high rate of 4 C, the electrode provides an enhanced discharge capacity of 53.2 mAh g^−1^ with the smaller applied potential range (1–1.85 V). These results indicate that the NMO/CNT composite is a promising cathode for aqueous sodium-ion batteries.

**Table 2:** Performance comparison of Na_*x*_MnO_2_-based cathodes for aqueous sodium-ion batteries

Materials	Reversible capacity (mAh g^−1^)	Cycle number	Current density	Applied potential range (V)	Reference
Na_4_Mn_9_O_18_	40	100th	4 C	0.5–2	[35]
Na_0.95_MnO_2_	40	1000th	4 C	1–2	[39]
NaMnO_2_	27.8	500th	5 C	0.5–1.8	[40]
Na_4_Mn_9_O_18_	40	–	0.1 C	0.2–0.9	[41]
Na_0.35_MnO_2_	43	500th	200 mA g^−1^	0–1	[42]
Na_4_Mn_9_O_18_/CNT	53.2	150th	4 C	1–1.85	This work

## Conclusions

In summary, the NMO/CNT particles have been successfully synthesized by spray-drying method. In addition, an aqueous sodium-ion battery using metallic Zn and NMO/CNT as the negative and positive electrodes, respectively, has been developed. The NMO/CNT electrode shows a larger discharge capacity of 96 mAh g^−1^ at 1 C rate and 53.2 mAh g^−1^ at 4 C rate even after 150 full cycles compared with the NMO electrode. The superior electrochemical performance of the NMO/CNT composite may arise from their spherical structure that can provide rapid transport and the addition of CNT that can enhance the conductivity of the composite. Overall, The NMO/CNT is a promising cathode material for the safe and efficient ASIBs.

## Abbreviations

ASIBs: Aqueous sodium-ion batteries
CV: Cyclic voltammetry
EIS: Electrochemical impedance spectroscopy
HR-TEM: High-resolution transmission electron microscope
LIBs: Lithium-ion batteries
NMO: Na_4_Mn_9_O_18_
NMO/CNT: Na_4_Mn_9_O_18_/carbon nanotube
NMP: *N*-methyl-2-pyrrolidone
PVDF: Polyvinylidene fluoride
SAED: Selected area electron diffraction
SEM: Scanning electron microscopy
TG: Thermo-gravimetric
XRD: X-ray powder diffraction
